# Study on the In-Laboratory Screening of Sandstone Grotto Patching Materials Based on Hydraulic Lime

**DOI:** 10.3390/ma18102192

**Published:** 2025-05-09

**Authors:** Shaoyun Zhang, Manli Sun, Zhipeng Li

**Affiliations:** 1 School of History and Culture, Northwest Minzu University, Lanzhou 730030, China; 2 School of Cultural Heritage, Northwest University, Xi’an 710127, China; sunml168@sohu.com; 3 Dunhuang Academy, Dunhuang 736200, China; lizp202212@163.com

**Keywords:** hydraulic lime, silica fume, patching materials, shrinkage rate, strength, sandstone grotto

## Abstract

This study explored the effect of compounding artificial hydraulic lime and silica fume for use as a sandstone grotto patching material. Different proportions of silica fume were added to hydraulic lime in lab tests, and their effects on the mortar’s physical and mechanical properties were studied. The results show that adding silica fume significantly increased the flexural and compressive strengths of the mortar and the shrinkage rate. A comparative analysis revealed that the comprehensive performance of the mortar reached the optimal state when the silica fume content was 10%. This met the strength requirements of repair materials for sandstone grottoes, as well as the control requirements regarding the shrinkage rate. Additionally, it demonstrated excellent weather resistance. This study’s results provide a scientific basis for the restoration of sandstone grottoes and the screening of an appropriate ratio of repair materials, which holds significant practical application value for the protection and reinforcement of stone relics.

## 1. Introduction

Recent research on the practical applications of natural hydraulic lime (NHL) have proven that it is an excellent material for the protection and reinforcement of ancient buildings and stone cultural relics [1,2,3,4]. At present, there are no known natural hydraulic lime deposits in China, and it must be imported from Europe if it needs to be used in large quantities. Therefore, Chinese scholars have attempted to prepare artificial hydraulic lime [5,6,7]. Zhang Shaoyun et al. successfully prepared artificial hydraulic lime (designated as K3) using potassium feldspar and limestone under high-temperature calcination [8,9]. K3 was selected as a protective material for the repair of sandstone grottoes to further explore its potential application in the conservation of stone cultural relics. 

When there are significant cracks at the top or noticeable depressions and erosion at the base of a rock mass in a grotto, patching techniques can reduce water infiltration along the cracks and prevent further erosion in the sunken areas. The patching material used in the restoration of stone cultural relics must have high strength [10]. Therefore, in addition to using K3 as the main binder, silica powder is incorporated to improve the strength of the mortar.

The use of silica fume in concrete has become widespread, primarily to improve the material’s properties; for example, to enhance its strength, durability, and impermeability. As a mineral admixture, silica fume effectively fills the gaps between cement particles, enhancing the microstructure of cement-based materials and improving the overall performance of concrete [11]. Additionally, silica fume has demonstrated significant benefits in improving concrete’s freeze resistance, chemical corrosion resistance, and high-temperature durability [12]. Research has shown that concrete containing silica fume achieves higher early strength and maintains superior structural stability and durability over time [13].

In recent years, the use of silica fume has been extended to the modification of hydraulic lime. Hydraulic lime, as a traditional building material, is mainly used in the conservation and strengthening of historical buildings. However, its performance in terms of compressive strength and durability is relatively weak [14]. The addition of silica fume can effectively enhance the mechanical properties and durability of hydraulic lime, thereby improving its applicability and performance in the restoration of historical buildings [15,16]. The addition of silica fume can improve the flexural and compressive strength of natural hydraulic lime mortar, but may also lead to an increase in the mortar’s shrinkage and water absorption. However, when silica fume was used to replace up to 20% of the hydraulic lime (NHL), it demonstrated an excellent performance [17]. Some studies have suggested that silica fume mortars have a relatively poor grouting ability but exhibit higher strength [18]. Most research indicates that silica fume, as a highly reactive pozzolanic material, reacts with calcium hydroxide to form calcium silicate gel, which enhances the strength of lime mortar.

There has been no research on the impact of mixing hydraulic lime K3 with silica fume on mortar’s physical properties. Therefore, this study compared the physical and mechanical properties of materials with different amounts of silica fume added and selected the optimal mix ratio for a material used to repair sandstone grottoes. The research findings provide a scientific basis for the restoration of sandstone grottoes and have significant practical value for the conservation and strengthening of stone cultural heritage.

## 2. Materials and Methods

### 2.1. Raw Materials

**Figure 1:**
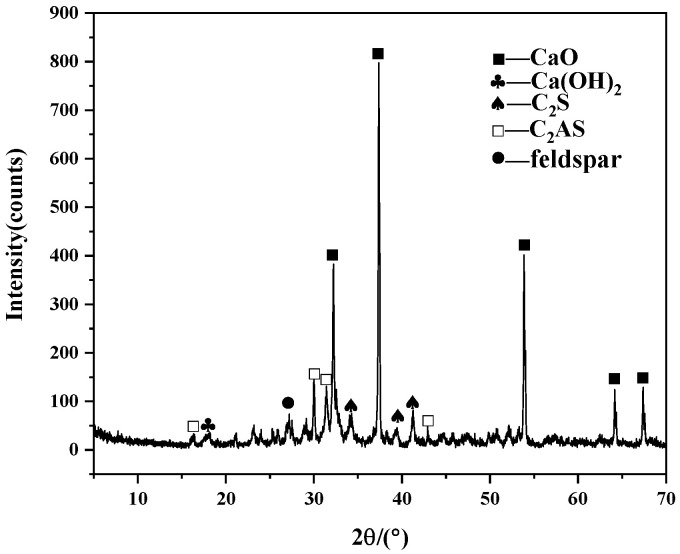
XRD pattern of K3 before slaking.

**Figure 2:**
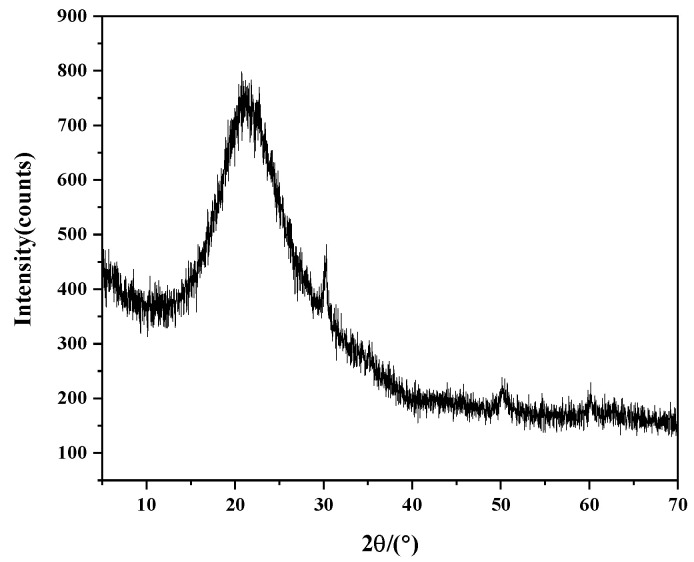
XRD pattern of silica fume.

In this experiment, hydraulic lime K3 was selected, which was obtained via the high-temperature roasting of a mixture of 30% potassium feldspar and 70% limestone by mass. Afterward, the K3 was slaked for 10 days and then crushed into powder with a particle size of approximately 80 µm. The silica fume was provided by Henan Yixiang New Material Technology Co., Ltd. (Hebi, China). The chemical compositions of both materials are shown in Table 1. Figure 1 and Figure 2 present the XRD patterns of the K3 before slaking and silica fume, respectively. The absence of a post-slaking XRD characterization of K3 is a major limitation of this study. However, subsequent mechanical performance tests and Zhao and Zhang et al.’s research [5,8] can indirectly demonstrate the reliability of the K3 material in cultural heritage conservation. The figures show that the main components of hydraulic lime K3 are calcium oxide, dicalcium silicate (C_2_S), and calcium aluminate silicate (C_2_AS). Both C_2_S and C_2_AS have hydraulic properties, which can provide early strength to the repair material, compensating for the slow development of strength in calcium oxide. The aggregates used in this experiment were sourced from Xiamen Aiso Standard Sand Co., Ltd. (Xiamen, China), which produces Chinese ISO standard sand, with a particle size range of 0.08–2 mm. This experiment used the Panalytical Axios FAST X-ray fluorescence spectrometer from Almelo, The Netherlands for the chemical composition analysis. The compositions of K3 and silica fume were tested using an X-ray diffraction instrument (XRD) (Rigaku RINT-2000, Tokyo, Japan), with Cu as the target, an operating voltage of 40 kV, a working current of 100 mA, a 2θ angle scan range of 5–70°, and a step rate of 0.02° per s.

### 2.2. Specimens’ Preparations

K3 was mixed with China ISO standard sand at a mass ratio of 1:1, and silica fume was added at concentrations of 5%, 10%, and 15% of the total mass, respectively. K3 was the binder material, standard sand was the aggregate, and silica fume was the admixture. The specimens were fabricated with a water–binder ratio of 0.5. The mortar, as a patching material for stone cultural relics, required a certain level of fluidity. Therefore, after conducting experiments, a water–binder ratio of 0.5 was chosen. The prismatic specimens with a size of 40 mm × 40 mm × 160 mm (width × depth × length) and cubic specimens of 70 mm × 70 mm × 70 mm were fabricated. The mortar mixture (K3, standard sand, and silica fume) was cast into molds according to the ratios specified in Table 2. These specimens were cured at 20 °C and 60% relative humidity (RH) for 28 days or 56 days. Subsequently, strength testing and elastic wave velocity measurements were conducted on the specimens. It is particularly necessary to point out the issue regarding the selection of curing conditions. This study was designed to simulate the actual environmental conditions of sandstone grottoes in Northwest China. This region features an arid climate (where the annual average humidity typically remains below 65%). Therefore, 20 °C and 60% relative humidity (RH) were selected to reflect real-world application scenarios, enabling the evaluation of K3-based material performance under near-natural conditions.

**Figure 3:**
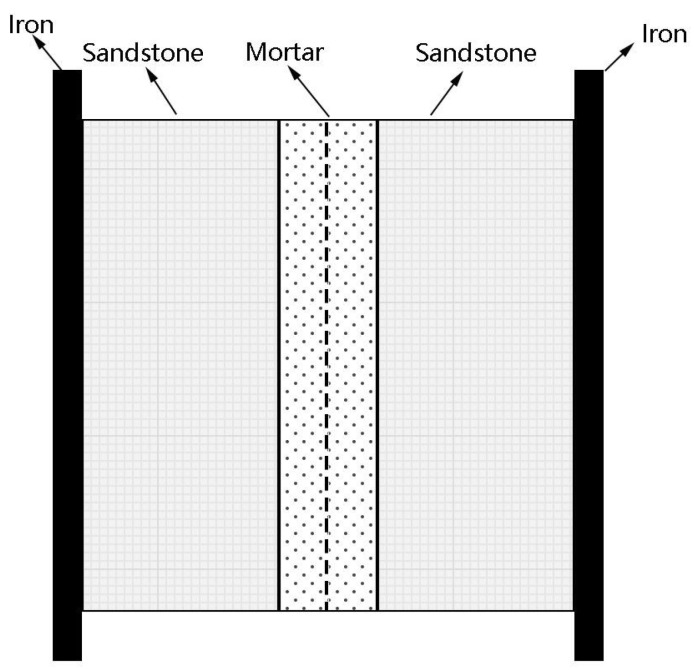
Schematic diagram of bond test.

The preparation of the sample used in the bond test is shown in Figure 3. First, a layer of lime mortar, approximately 5 mm thick, was applied to the bond surfaces of two rock pieces. The bond surfaces were then pressed together. The rock dimensions were 5 cm × 5 cm × 2 cm, with a bond area of 25 cm^2^. After the test samples were prepared, they were cured for 56 days. Once cured, 3 mm thick iron plates were attached to both sides of the sample using epoxy resin to secure the pulling clamps of the electronic tensile testing machine, and a tensile test was performed. A set of three specimens was fabricated for the tensile test.

### 2.3. Test Methods

#### 2.3.1. Mechanical Properties Test

The prismatic specimens were subjected to the center-point loading flexural test. The test was conducted at a fixed rate of 0.1 mm/min using an Instron Testing Machine. The prismatic specimens were tested until they were broken into two halves, and their flexural strength was computed. A set of three specimens was fabricated for the flexural test. In addition, both portions from each broken prismatic specimen were used for compressive strength testing, as per the CEN guidelines. EN1015-11:1999/A1:2006 [19]. The one-half-prismatic specimen had a length of no less than 65 mm and was free of a cracked, chipped surface or other obvious defects. The compressive strength of the jig section of each one-half-prismatic specimen was computed by dividing the maximum imposed load by 1600 mm^2^ of the testing area (the jig section area).

The shrinkage was determined using a retractometer per ASTMC 1148-92a:2008 [20]. The elastic wave velocities were tested using a non-metal sonic apparatus (RSM-SY5 mode, Zhongke Zhichuang, Wuhan, China) with a frequency of 50 kHz. 

#### 2.3.2. Durability Test

Accelerated aging tests can better reflect samples’ durability; therefore, all durability tests were conducted using lime mortar specimens cured for 56 d.

(1) Water stability test

The specimens, which had aged for 56 days, were submerged in water at a temperature of 20 °C for 24 h. After being removed from the water, the surfaces of the specimens were dried. Subsequently, the compressive and flexural strength tests were conducted promptly.

(2) Soundness test

The specimens, which had aged for 56 days, were immersed in a saturated Na_2_SO_4_ solution for 20 h. Subsequently, the specimens were removed from the solution and baked at 105 °C for 4 h. After completing five cycles of this test, the specimens’ strength was tested.

(3) Alkali resistivity test

After aging for 56 days, the specimens were soaked in a 2% NaOH solution for 12 h. Then, the specimens were removed from the solution and baked at 105 °C for 4 h. Subsequently, the specimens’ strength was tested.

(4) Freeze–thaw cycle test

After reaching 56 days of age, the specimens were stored in a refrigerator and frozen to below −30 °C for 12 h. Following this, they were subjected to a curing process at 20 °C and 70% relative humidity for 12 h. Once eighteen cycles were completed, the specimens’ strength was tested.

(5) Temperature and humidity cycle test

The specimens aged 56 days were heated for 12 h at 100 °C. Then, the specimens were cured at 20 °C and 70% RH for 12 h. After eighteen cycles, the specimens’ strength was tested.

## 3. Results

### 3.1. Shrinkage Rate

**Figure 4:**
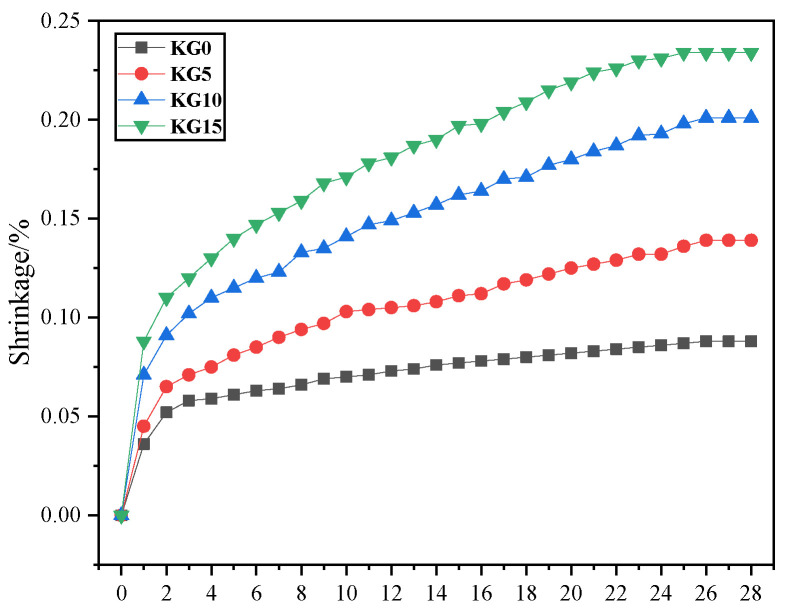
Shrinkage rates of the specimens with different dosages of silica fume.

A smaller shrinkage rate is conducive to the patching and reinforcement of stone relics so that the slurry and rock can be closely conglutinated to achieve the ideal reinforcement effect [21]. Figure 4 illustrates the changes in the shrinkage rates of the specimens containing varying amounts of silica fume at different curing ages. The figure shows that from days 0 to 3, all four types of specimens experienced rapid shrinkage, mainly attributed to the early solidification of the paste and the natural evaporation of moisture. After the third day, the shrinkage of the specimens began to decelerate. Between days 14 and 21, the rate of increase in the shrinkage rates for these specimens further decreased. At 14 days, the shrinkage rates of the four specimen types (KG0, KG5, KG10, and KG15) reached 82%, 76%, 77%, and 80% of their respective 28-day shrinkage values, respectively. At 21 days, the shrinkage rates of the four specimen types (KG0, KG5, KG10, and KG15) reached 93%, 90%, 90%, and 94% of their respective 28-day shrinkage values, respectively. By day 26, the shrinkage rates of all four specimens had stabilized, showing no further changes.

As the silica fume content in the mortar increased, the shrinkage rate of the test specimens also rose. This was mainly because the self-drying property of silica fume increased the volume shrinkage of the mortar material itself, which was almost the same as that of the dry shrinkage. Additionally, the reduction in the water–binder ratio increased the mortar’s self-shrinkage [22]. Therefore, a higher silica fume content led to increased shrinkage in the composite material, which contrasts with the previous literature reporting that silica fume improves the shrinkage properties of hydraulic lime mortar [23]. In addition, the free water within the mortar was restrained by the silica fume, slowing down the hydration reaction process in the hydraulic lime, which inhibited the specimens’ shrinkage. Therefore, the more silica fume is added, the greater the shrinkage. The shrinkage rates of the four specimens at 28 days were as follows: 0.23% for KG15, 0.20% for KG10, 0.14% for KG5, and 0.09% for KG0. From the perspective of the shrinkage rate alone, the shrinkage rates of the four lime mortars were relatively low, meeting the requirements for sandstone grotto patching materials.

### 3.2. Slurry Fluidity

**Figure 5:**
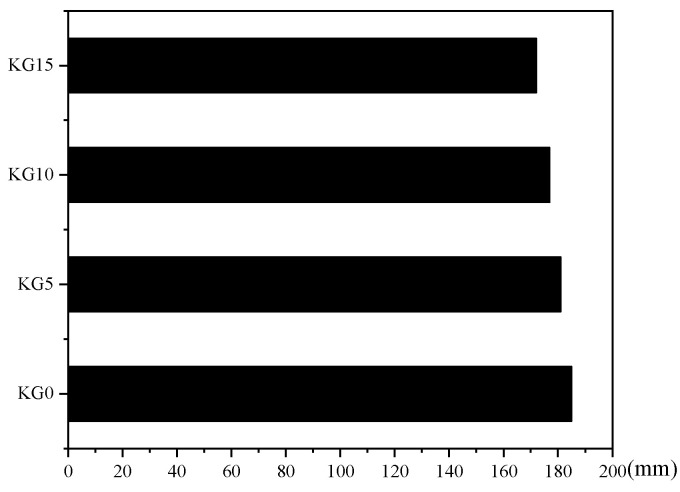
Slurry fluidity with different dosages of silica fume.

Slurry fluidity is a crucial factor in patching materials for stone cultural relics. Excessive fluidity can lead to weaker adhesion during repairs, while insufficient fluidity might result in the existence of areas within small cracks where the slurry cannot reach. Figure 5 illustrates that the slurry fluidity decreased as more silica fume was added, although the reduction was not substantial. This is primarily because silica fume particles are very fine. When a small amount was added to the slurry, these particles filled the gaps between coarser lime particles, optimizing the particle size distribution. Additionally, they displaced some of the water used for filling, which enhanced the slurry’s fluidity. Therefore, although the difference in the silica fume content affected the slurry’s fluidity, the four mixtures’ fluidities did not significantly differ. The fluidity values were as follows: 185 mm for KG0, 181 mm for KG5, 177 mm for KG10, and 172 mm for KG15. The fluidity of all these mixtures met the requirements for patching.

### 3.3. Flexural and Compressive Strength of Mortars

**Figure 6:**
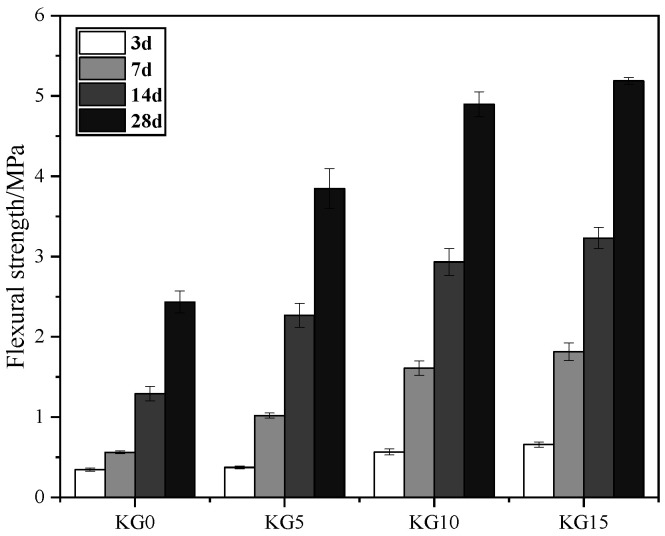
Flexural strength of the specimens with different dosages of silica fume.

**Figure 7:**
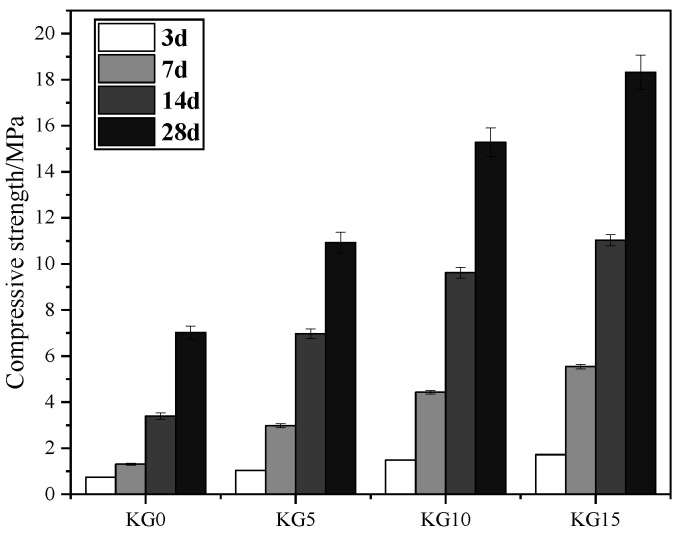
Compressive strength of the specimens with different dosages of silica fume.

As shown in Figure 6 and Figure 7, the strength of the four specimens varied with different curing times. The strength gradually increased with the incorporation of silica fume and an increase in its content. When 5% silica fume was added, the 28-day flexural strength and compressive strength of KG5 were 3.85 MPa and 10.93 MPa, respectively. When 10% silica fume was added, the 28-day flexural strength and compressive strength of KG10 were 4.90 MPa and 15.28 MPa, respectively. When 15% silica fume was added, the 28-day flexural strength and compressive strength of KG15 were 5.19 MPa and 18.32 MPa, respectively.

Analyzing the 28-day strength data, the silica fume content rose from 5% to 10%, and the flexural strength and compressive strength exhibited significant growth rates of 27.27% and 39.80%, respectively. Further increasing the silica fume content from 10% to 15% resulted in reduced growth rates of 5.59% for flexural strength and 19.90% for compressive strength. This indicates that the strength gains were more substantial when the silica fume content was increased from 5% to 10%. Although the strength continued to improve with a silica fume content of 15%, the rate of this improvement was slower. This phenomenon may be attributed to two primary factors: First, the excessive silica fume content reduced the slurry’s fluidity, compromising the uniformity of the pozzolanic reaction and hindering interfacial structure optimization. Second, the localized accumulation of surplus silica fume particles within the slurry matrix may have obstructed the homogeneous distribution of hydration products, thereby suppressing the formation of a fully dense microstructure. These combined effects limited further enhancements in the mechanical performance despite the continued pozzolanic activity at higher silica fume dosages. Overall, the more silica fume was added, the greater its later strength. This was mainly because the silica fume had very strong pozzolanic activity, which reacted with the calcium hydroxide in the hydraulic lime to form hydrated calcium silicate gel, filling the gaps between the stone particles, improving the interface structure and adhesion, and forming a dense structure, thereby significantly increasing the strength of the lime mortar material.

### 3.4. Bond Strength

The bond test’s main purpose was to test the bond strength between the repair material and the body of the restored stone cultural relic, i.e., to detect the compatibility effect between the mortar and the body of the restored stone cultural relic. The quality of the bonding performance of the patching material will significantly impact the fracture surface of the stone cultural relic. If the patching material has poor compatibility with the stone cultural relic, it will peel off from the stone cultural relic. Therefore, the bond test is used to better evaluate the compatibility between the protective material and the cultural relic. The bond strength is an important indicator for measuring the degree of bonding between repair materials and a rock mass. A higher bond strength is more beneficial for the adhesion of repair materials to the sandstone matrix interface, improving the stability of the rock mass after repair.

**Figure 8:**
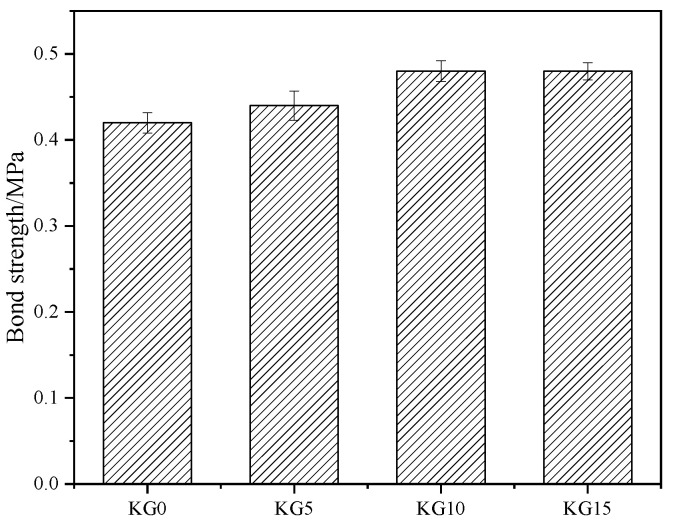
Bond strength of samples silica fume.

The bond strength of the four specimens was tested at 56 days, using sandstone collected from the North Grottoes in Gansu, China. The results are shown in Figure 8. As the amount of silica fume increased, the bond strength of the mortar stone body also gradually increased. However, the growth rate was not as significant as the increase in flexural strength and compressive strength. The analysis indicates that observed results were likely due to the sandstone’s inherently low strength, which tended to fracture internally during the tensile testing process. This internal failure led to relatively minor variations in the bond strength among the four stone specimens. Nonetheless, the bond strength of all four materials surpassed 0.4 MPa, thereby meeting and exceeding the bond strength requirements outlined in the relevant literature [24].

### 3.5. Wave Velocity and Age Relationship

**Figure 9:**
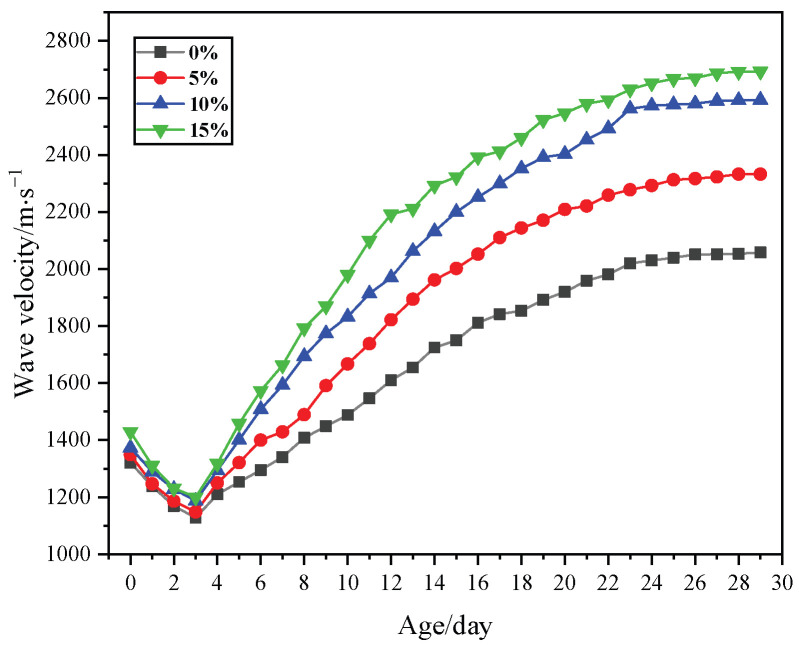
Diagram showing the relationship between the age of the stone body, different dosages of wave velocity, and different dosages of silica fume.

The wave velocity is influenced by various factors, such as the porosity of the test block, the water content, and the internal aggregate distribution. It can reflect the relationship between the internal structure and the strength changes in materials at different ages. The relationship between the test block age and wave velocity after adding different amounts of silica fume is shown in Figure 9. The following conclusions can be drawn from the figure:

(1) The changes in wave velocity for the four test blocks were similar. The wave velocity decreased from days 1 to 3, mainly due to the presence of a large amount of liquid water and calcium silicate colloids in the blocks at the early stages of the test, causing the attenuation and scattering of the wave velocity. After day 3, the wave velocity rapidly increased, with the fastest growth rate from days 3 to 14. After day 14, the growth rate slowed down. However, the wave velocity of the test blocks maintained a relatively fast growth rate from days 14 to 24. After day 24, the wave velocity slowly increased and stabilized.

(2) After day 3, the sample with 15% silica fume addition showed the fastest increase in wave velocity, followed by the samples with 10% and 5% silica fume additions, respectively. The sample without the silica fume addition had the slowest wave velocity increase. This was mainly because the silica fume reacted with the calcium hydroxide in the hydraulic lime via a pozzolanic reaction, rapidly forming solid bridges within the sample and allowing the wave velocity to quickly propagate along the solid particles. In contrast, the sample without silica fume addition primarily underwent the hydration of calcium silicate and the carbonation of calcium hydroxide, which are slower processes than the pozzolanic reaction, resulting in a slower increase in the wave velocity.

(3) The increase in the strength of the four test blocks was generally in line with the changes in the wave velocity. The test block with a 15% addition of silica fume demonstrated the highest strength and the most rapid increase in the wave velocity, whereas the test block without any silica fume showed the lowest strength. The strength of the test blocks rapidly increased from days 3 to 14. Although it continued to increase thereafter, the growth rate slowed down. From days 14 to 28, the strength still increased, but the growth rate was significantly slower.

### 3.6. Specimens’ Durability

Considering the age-related strength, shrinkage rate, and workability of the sample test blocks, KG10 was deemed to be a more suitable option for the restoration of sandstone grottoes. Strength tests on KG10 were carried out under various conditions, including water stability, soundness, resistance to alkalis, freeze–thaw cycles, and temperature and humidity variations.

#### 3.6.1. Water Stability Test

**Figure 10:**
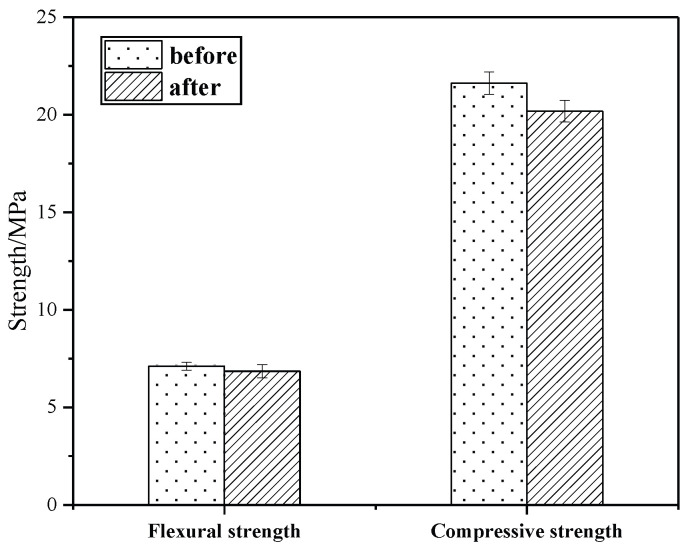
Strength comparison when immersed in water.

Figure 10 shows the compressive and flexural strengths of the specimen before and after the tests. The flexural strength and compressive strength of KG10 slightly decreased after being immersed in water, but its appearance and surface remained intact and unchanged. The calculated softening coefficient was K = 0.93, indicating that the test block had very good water resistance and could effectively seal against water after repair.

#### 3.6.2. Soundness Test

**Figure 11:**
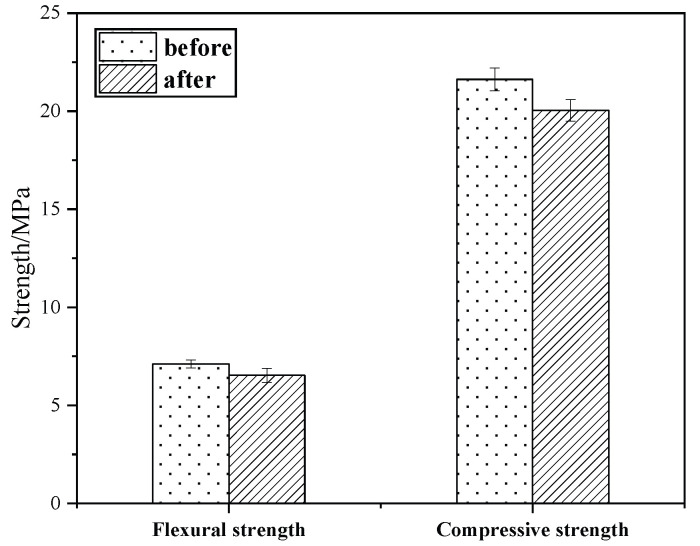
Strength comparison in soundness experiment.

The salt crystallization of sodium sulfate is an important factor in the deterioration of stone relics, as this damages the reinforcement material [25]. Figure 11 shows the compressive and flexural strengths before and after the test for KG10. Following five cycles of immersion in sodium sulfate solution, the KG10 test specimen maintained its integrity without any visible damage to its appearance or surface. KG10’s strength showed a slight decline post-immersion, with minimal reductions in the flexural and compressive strength of 8.16% and 7.31%, respectively. These results suggest that KG10 possesses excellent stability. Moreover, it offers significant resistance to salt erosion when used as a restoration material for sandstone grottoes.

#### 3.6.3. Alkali Resistivity Test

**Figure 12:**
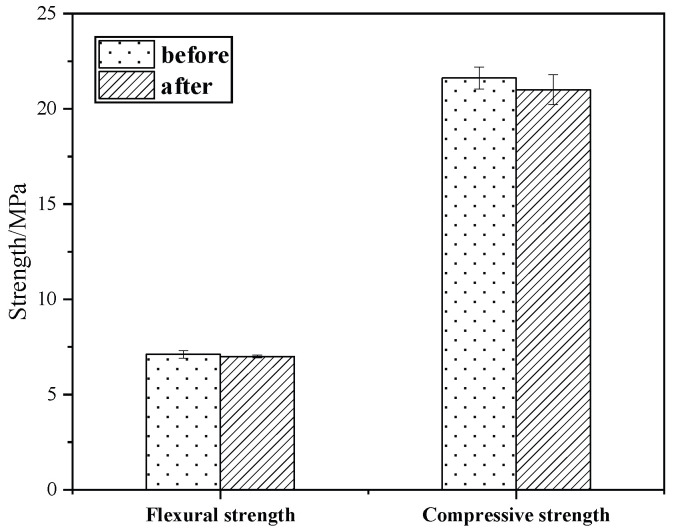
Strength comparison in alkali resistivity experiment.

KG10’s appearance remained intact, without any changes, after being immersed in sodium hydroxide solution and baked. Figure 12 shows that KG10’s flexural strength remained almost unchanged after immersion in the alkaline solution, while its compressive strength slightly decreased. The loss rates of flexural strength and compressive strength were 1.55% and 2.87%, respectively. The tests proved that the KG10 mortar material had strong alkali resistance, and the material exhibited excellent resistance to salt and alkali corrosion after being used to repair sandstone grottoes.

#### 3.6.4. Freeze–Thaw Cycle Test

**Figure 13:**
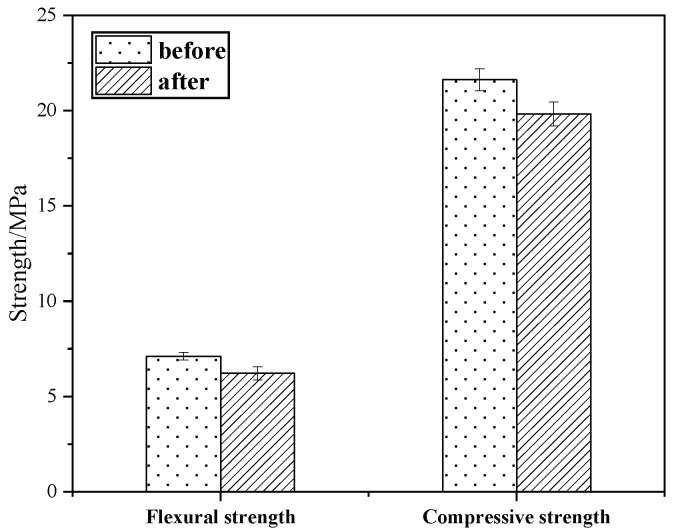
Strength comparison in freeze–thaw cycle.

Many stone relics are preserved in Northwest China, which is colder in winter. Therefore, the freeze–thaw cycle is a necessary test to detect the cold resistance of protective materials. The KG10 test block showed no changes in appearance or surface and no mass loss after undergoing 18 freeze–thaw cycles. A comparison of the KG10 test block’s strength before and after the freeze–thaw cycles is presented in Figure 13. After the freeze–thaw test, both the flexural and compressive strength of KG10 decreased, but the decline was not significant. The loss rates of flexural and compressive strength were 12.52% and 8.28%, respectively. According to the testing requirements of the freeze–thaw test, the strength loss rate of KG10 was below 25%, indicating that it has good freeze–thaw resistance.

#### 3.6.5. Temperature and Humidity Cycle Test

**Figure 14:**
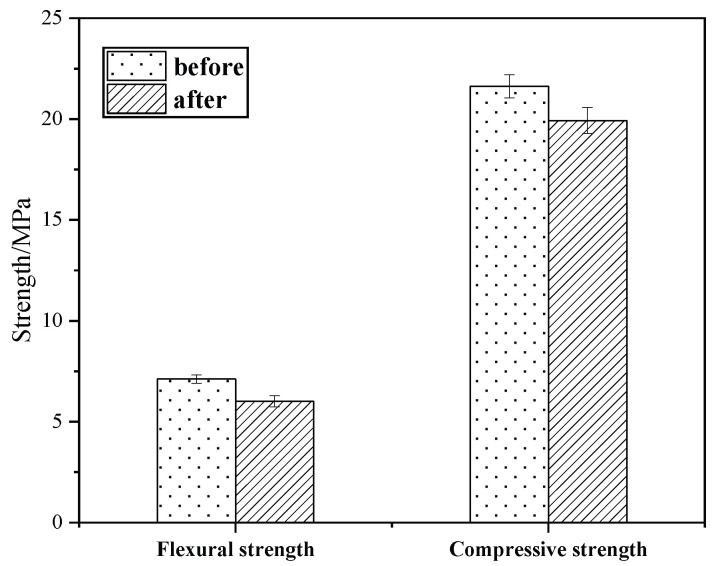
Strength comparison over temperature and humidity cycles.

Temperature and humidity changes in the environment can significantly impact the preservation of cultural relics. Therefore, an accelerated aging test temperature and humidity cycle could effectively evaluate KG10’s durability. The KG10 test block remained intact without any cracks after undergoing 18 temperature and humidity cycles. As shown in Figure 14, both the flexural and compressive strength of KG10 decreased after the temperature and humidity cycles, with loss rates of 15.47% and 7.86%, respectively. Therefore, fluctuations in environmental temperature and humidity will not cause significant damage to KG10 (i.e., it demonstrates resistance to temperature and humidity cycles), making it suitable for the protection and restoration of grottoes in the northern region of China, which feature a large temperature difference between day and night.

## 4. Discussion

This study demonstrated that incorporating silica fume into artificial hydraulic lime (K3) significantly enhances the mechanical properties of mortars for sandstone grotto restoration. KG15 exhibited the highest shrinkage rate (0.23% at 28 days). While this underscores the role of silica fume in enhancing strength, excessive shrinkage risks compromising interfacial bonding. KG10 emerged as a balanced candidate, mitigating shrinkage escalation while retaining mechanical gains. All mixes maintained a fluidity of >170 mm, meeting the patching requirements. The pozzolanic reaction of silica fume with K3’s Ca(OH)_2_ drove strength gains. KG10 achieved a 39.8% compressive strength increase (15.28 MPa) compared to KG5, whereas KG15’s slower growth signaled diminishing returns, likely due to localized particle accumulation and hydration constraints. The bond strength plateaued near 0.4 MPa across the mixes, limited by the sandstone substrate’s inherent low strength. Nonetheless, KG10’s compatibility with the rock matrix ensured cohesive failure within the substrate, validating its suitability for structural adhesion. The optimal performance was achieved with 10% silica fume (KG10).

KG10 demonstrated exceptional durability across aggressive environmental tests. In the water stability trials, it exhibited a softening coefficient of 0.93 with a minimal strength loss (<7%), confirming its resistance to moisture-induced degradation. Under salt and alkali exposure, the material maintained robust chemical stability, showing strength declines of less than 8%, a critical attribute for resisting sulfate and alkaline erosion common in grotto environments. Furthermore, KG10 endured freeze–thaw cycles and temperature fluctuations, with strength losses below 15%, meeting the stringent durability thresholds and underscoring its suitability for the harsh climatic conditions of Northwest China, characterized by extreme seasonal temperature variations. These results collectively validate KG10’s resilience as an ideal patching material for sandstone grotto conservation.

However, unlike studies on natural hydraulic lime (NHL) that focused on higher silica fume replacements (up to 20%) [17], this work highlights a critical threshold (10%) for K3-based systems, beyond which the shrinkage escalation outweighs strength gains.

KG10’s durability likely stems from its dense microstructure, formed by the synergistic hydration of K3’s hydraulic phases (C_2_S and C_2_AS) and silica fume’s reactive SiO_2_, which generates additional calcium silicate hydrate (C-S-H). This mechanism mirrors the observations in NHL5 and K3 mortars, where hydraulic phases dominate and enhance carbonation resistance [8].

This study had several limitations. First, the experiments were conducted under controlled laboratory conditions, which may not fully replicate the complex environmental stressors (e.g., wind erosion, biological colonization, and cyclic wet–dry conditions) faced by sandstone grottoes in situ. Second, the maximum silica fume content tested was 15%; higher proportions might reveal additional performance trends or trade-offs. Third, although the stability of the microstructure was indirectly reflected through the integration of the literature, elastic wave velocity measurements, and prolonged aging tests, this study exhibits limitations in its characterization methods (e.g., the absence of SEM/MIP analyses for microstructural investigation). Fourth, the absence of post-slaking XRD characterization of K3 is a major limitation of this study.

Future studies should prioritize field trials to validate KG10’s long-term stability in real grotto environments. Additionally, exploring additives, such as fibers or polymers, could mitigate shrinkage without compromising strength. The addition of nanomaterials, such as nano-silica, to K3 to enhance its mechanical properties is another future research direction [26].

## 5. Conclusions

Comparing the four samples, the greater the amount of silica fume that was added, the higher the shrinkage rate. The fluidity of the four sample slurries differed little, at approximately 180 mm, which meets the requirements for the fluidity of the repair material. Both the flexural and compressive strength of the test blocks increased as the silica fume was added. The bond strength also increased with increasing silica fume content, and the difference in the bond strength between the test blocks with different amounts of silica fume was not significant. The wave velocity of the test blocks showed a trend of first decreasing and then increasing, and then it rapidly increased with an extension of the curing time. The test block with the highest silica fume content had the greatest wave velocity, which was consistent with the increase in strength.

Higher strength is beneficial for a grotto repair material, helping it to resist the impact of the external environment and thereby reducing the damage to the grotto. Furthermore, a slurry with lower shrinkage and suitable fluidity is advantageous for the bonding between the repair material and the rock mass interface. Therefore, considering factors such as the shrinkage rate, fluidity, and strength, when the cement–sand ratio is 1:1, the water–binder ratio is 0.5, and the silica fume content is 10%, this material can be used as a repair material for sandstone grottoes. It should be noted that although direct microstructural characterization data are lacking, based on mechanical properties, wave velocity variations, and analogy with the SEM results of hydraulic lime in Reference [16], it can be reasonably inferred that a continuous C-S-H network structure formed in the K3-based material with a 10% silica fume addition.

After weather resistance testing, KG10 exhibited good water resistance, resistance to salt and alkali corrosion, and good freeze–thaw resistance. It also showed robust resistance to fluctuations in environmental temperature and humidity. However, it should be noted that while KG10 demonstrated outstanding durability under accelerated aging conditions, its long-term durability still requires further validation through in situ environmental monitoring and extended cycle testing.

## Figures and Tables

**Table 1:** Chemical compositions of K3 and silica fume (mass/%).

Composition	SiO_2_	Al_2_O_3_	Na_2_O	K_2_O	CaO	MgO	Fe_2_O_3_	Others	LOI
K3	27.90	5.85	0.63	2.87	49.27	1.13	0.26	0.10	11.99
Silica fume	95.73	0.20	0.00	0.07	0.12	0.03	0.25	3.58	0.00

**Table 2:** Sample ratios and curing conditions.

Sample No.	Silica Fume Content	Water–Binder Ratio	Curing Conditions
KG0	0	0.5	20 °C RH 60%
KG5	5%	0.5	
KG10	10%	0.5	
KG15	15%	0.5	

## Data Availability

The original contributions presented in this study are included in the article. Further inquiries can be directed to the corresponding author.

## References

[1] Maravelaki-Kalaitzaki P., Bakolas A., Karatasios I. (2004). Hydraulic lime mortars for the restoration of historic masonry in Crete. Cem. Concr. Res..

[2] Lanas J., Bernal J.L.P., Bello M.A. (2004). Mechanical properties of natural hydraulic lime-based mortars. Cem. Concr. Res..

[3] Zhou X., Hu Y., Wang J.H., Dai S.B. (2011). Study on the application of hydraulic lime in the consolidation and protection of Huashan rock paintings. Sci. Conser. Arc..

[4] Bras A., Henriques F.M.A., Cidade M.T. (2010). Effect of environmental temperature and fly ash addition in hydraulic lime grout behavior. Constr. Build. Mater..

[5] Zhao L.Y., Li L., Li Z.X. (2011). Research on two traditional silicate materials in China’s ancient building. J. Inorg. Mater..

[6] Shen X.F., Xue Q.H., Xu L.L., Shi Z.W., Zhang H. (2013). Research of the feasibility to prepare the natural hydraulic limes from the lead and zinc mine tailing. Bull. Chin. Ceram. Soc..

[7] Wang L.L., Liu Z., Wang D.M., Jiang Q.K., Zhang C. (2019). Process optimization and properties of natural hydraulic lime from marlite. Bull. Chin. Ceram. Soc..

[8] Zhang S.Y., Sun M.L., Guo Q.L., Zhao L.Y., Li Z.P. (2023). Study on the mechanical properties and durability of hydraulic lime mortars based on limestone and potassium feldspar. Appl. Sci..

[9] Dunhuang Academy (2022). A Kind of Hydraulic Lime Material, Preparation Method and Application: China, ZL202110885483.8.

[10] Zhang B.J., Wang X. (2000). Stone bonding and repair technology. Stone.

[11] Ahmad S., Al-Amoudi O.S.B., Khan S.M.S., Maslehuddin M. (2022). Effect of silica fume inclusion on the strength, shrinkage and durability characteristics of natural pozzolan-based cement concrete. Case Stud. Constr. Mater..

[12] Buller A.S., Abro F.-u.-R., Ali M. (2024). Effect of silica fume on fracture analysis, durability performance and embodied carbon of fiber-reinforced self-healed concrete. Theor. Appl. Fract. Mech..

[13] Lachemi M., Li G., Tagnit-Hamou A., Aitcin P.-C. (1998). Long-term performance of silica fume concretes. Concr. Int..

[14] Xu S.Q., Ma Q.L., Wang J.L. (2018). Grouting performance improvement for natural hydraulic lime-based grout via incorporating silica fume and silicon-acrylic latex. Constr. Build. Mater..

[15] Xu S.Q., Ma Q.L., Wang J.L. (2018). Combined effect of isobutyltriethoxysilane and silica fume on the performance of natural hydraulic lime-based mortars. Constr. Build. Mater..

[16] Xu S.Q., Wang J.L., Ma Q.L., Wang L.L., Zhang T. (2023). Effect and mechanism of two different types of waterproof admixtures and silica fume on the hydration and mechanical properties of natural hydraulic lime. Case Stud. Constr. Mater..

[17] Zong X., Zhang Q.Z. (2022). Organic/inorganic composite modification of natural hydraulic lime. J. Chongqing Univ. Sci..

[18] Luis G.B. (2022). Performance of silica fume-based geopolymer grouts for heritage masonry consolidation. Crystals.

[19] (2006). Methods of Test for Mortars for Masonry. Part 11: Determination of Flexural and Compressive Strength of Hardened Mortar.

[20] (2008). Standard Test Method for Measuring the Drying Shrinkage of Masonry Mortar.

[21] Li Z.X., Zhao L.Y., Li L. (2011). On new fracture grouting material for conglomerate grottoes rock. Dunhuang Res..

[22] Yang Q.G., Yuan P., Chen D.W. (2009). Experimental study on the influence of silica fume on the mechanical properties of medium strength concrete. Traffic Stand..

[23] Xu S.Q., Wang L.L., Ma Q.L. (2017). Hydration of natural hydraulic lime under different carbonation conditions. Sci. Conser. Arc..

[24] Li L., Zhao L.Y. (2015). Study on Ancient Chinese Calcareous Materials.

[25] Wang J.F., Yan G.S., Yang S.L. (2010). Distribution of soluble salts in the cliff strata of the Mogao Grottoes. Hydrogeol. Eng. Geol..

[26] Luo K., Li J., Han Q., Lu Z.Y., Deng X., Hou L., Niu Y.H., Jiang J., Xu X.Y., Cai P. (2020). Influence of nano-SiO_2_ and carbonation on the performance of natural hydraulic lime mortars. Constr. Build. Mater..

